# Detecting heterogeneity in and between breast cancer cell lines

**DOI:** 10.1186/s41236-020-0010-1

**Published:** 2020-02-03

**Authors:** Yang Shen, B. U. Sebastian Schmidt, Hans Kubitschke, Erik W. Morawetz, Benjamin Wolf, Josef A. Käs, Wolfgang Losert

**Affiliations:** 1 Institute for Physical Science and Technology, University of Maryland, College Park, MD 20742 USA; 2 Peter Debye Institute for Soft Matter Physics, Leipzig University, Linnéstr. 5, 04103 Leipzig, Germany; 3 Leipzig University Medical Center, Department of Obstetrics and Gynecology, Liebigstr. 20a, 04103 Leipzig, Germany

**Keywords:** Cancer, Heterogeneity, Single-cell, MDA-MB-231, MCF-10A, MDA-MB-436

## Abstract

**Background:**

Cellular heterogeneity in tumor cells is a well-established phenomenon. Genetic and phenotypic cell-to-cell variability have been observed in numerous studies both within the same type of cancer cells and across different types of cancers. Another known fact for metastatic tumor cells is that they tend to be softer than their normal or non-metastatic counterparts. However, the heterogeneity of mechanical properties in tumor cells are not widely studied.

**Results:**

Here we analyzed single-cell optical stretcher data with machine learning algorithms on three different breast tumor cell lines and show that similar heterogeneity can also be seen in mechanical properties of cells both within and between breast tumor cell lines. We identified two clusters within MDA-MB-231 cells, with cells in one cluster being softer than in the other. In addition, we show that MDA-MB-231 cells and MDA-MB-436 cells which are both epithelial breast cancer cell lines with a mesenchymal-like phenotype derived from metastatic cancers are mechanically more different from each other than from non-malignant epithelial MCF-10A cells.

**Conclusion:**

Since stiffness of tumor cells can be an indicator of metastatic potential, this result suggests that metastatic abilities could vary within the same monoclonal tumor cell line.

## Background

Recognized as early as 1958 (Huxley 1958), genetic heterogeneity is a well-established phenomenon in tumor cells, especially during metastatic stages (Torres et al. 2007; Park et al. 2010; Patel et al. 2014; Alizadeh et al. 2015). Studies have shown that cells from a single cancer typically contain multiple genetically distinct subgroups (Cleary et al. 2014; Meacham and Morrison 2013; Gay et al. 2016; Marusyk and Polyak 2010). Such high level of heterogeneity contributes to the reason why cancer is hard to cure (McGranahan and Swanton 2017; Mann et al. 2016; Koren and Bentires-Alj 2015). However, to-date the reason and extent of tumor cell heterogeneity is still not well-understood (Alizadeh et al. 2015). Two main theories have been proposed to explain the origin of tumor cell heterogeneity: the existence of cancer stem cells (Magee et al. 2012) and clonal evolution (McGranahan and Swanton 2017). These two theories try to explain the heterogeneity in ecological and evolutional aspects, respectively, and evidence exists for each theory (Shackleton et al. 2009). Furthermore, new insight in gene regulatory networks provides a framework for explaining the broad heterogeneity without the need of excessive mutational activity (Huang 2012a; Huang 2013; Huang 2012b). Variations in gene expression lead to molecular variations which in turn affect cellular shape and function.

Another well-established phenomenon associated with tumors are changes in cellular stiffness. Cells actively structure and regulate the different elements of the cytoskeleton, the main contributor of cellular stiffness and compliance (Huber et al. 2013). In fact, different components of the cytoskeleton contribute to different structural and mechanical tasks, e.g. actin contributes to cell elasticity in response to small strains while microtubules affect responses to large strains (Lautenschlager et al. 2009; Kubitschke et al. 2017). The mechanics of cells has been studied with multiple experimental tools (Kubitschke et al. 2018; Pawlizak et al. 2015), including atomic force microscopy (AFM) (Hayashi and Iwata 2015), quantitative deformability cytometry (q-DC) (Nyberg et al. 2017), real-time deformability cytometry (Mietke et al. 2015; Otto et al. 2015), microfluidic optical cell stretchers (Farzbod and Moon 2018), and hydrodynamic flow stretchers (Dudani et al. 2013; Gossett et al. 2012). Since metastasis is responsible for more than 90% of cancer fatality (Wirtz et al. 2011; Mehlen and Puisieux 2006; Taketo 2011), great effort has been made to study the mechanical properties of metastatic tumor cells and to understand how mechanical properties of tumor cells affect their metastatic ability. A number of studies have found that metastatic tumor cells are softer than their non-metastatic counterparts as well as normal cells (Lekka et al. 2012; Plodinec et al. 2012; Swaminathan et al. 2011). In addition, studies have suggested the potential of using mechanical properties as a biomarker of metastasis (Xu et al. 2012) and for cancer diagnosis (Remmerbach et al. 2009).

In this paper we take first steps to link these two phenotypes of metastatic tumor cells – changes in cell heterogeneity and cell stiffness. Though most cell mechanics studies are carried out at the single-cell level, analysis and interpretation of data is generally confined to averages, thus omitting heterogeneity as an important aspect of the metastatic phenotype. Prior work (Plodinec et al. 2012; Kiessling et al. 2013) has yielded important hints that mechanical properties are in fact heterogeneous – the measured distributions for the viscoelastic properties of cells, even in a single cell line, are not Gaussian indicating that various mechanical phenotypes are present, for instance, represented by outliers of the usual long-tailed distributions.

In this paper, we use a microfluidic optical cell stretcher to measure and contrast mechanical properties of single cells from three epithelial cell lines: MCF-10A, MDA-MB-231 and MDA-MB-436, and we use the heterogeneity of the cell mechanical properties of each cell line to contrast the different phenotypes. These three cell lines represent a well-established breast cancer cell panel. MCF-10A is a non-tumorigenic epithelial cell line while MDA-MB-436 and MDA-MB-231 are breast carcinoma cell lines with a mesenchymal-like metastatic phenotype. With single cell data analysis, we show that heterogeneity of cellular stiffness exists both within and between cell lines. In particular, we observe two groups of MDA-MB-231 cells. Cells in one of the groups are significantly softer than cells in the other. In addition, we find that although MDA-MB-231 and MDA-MB-436 are both triple-negative breast cancer cell lines (i.e. they do not express estrogen receptors, progesterone receptors nor human epidermal growth factor receptor [HER]2) with metastatic tendency, they are rather distinct from each other compared to the nonmalignant cell line MCF-10A.

## Results

**Fig. 1:**
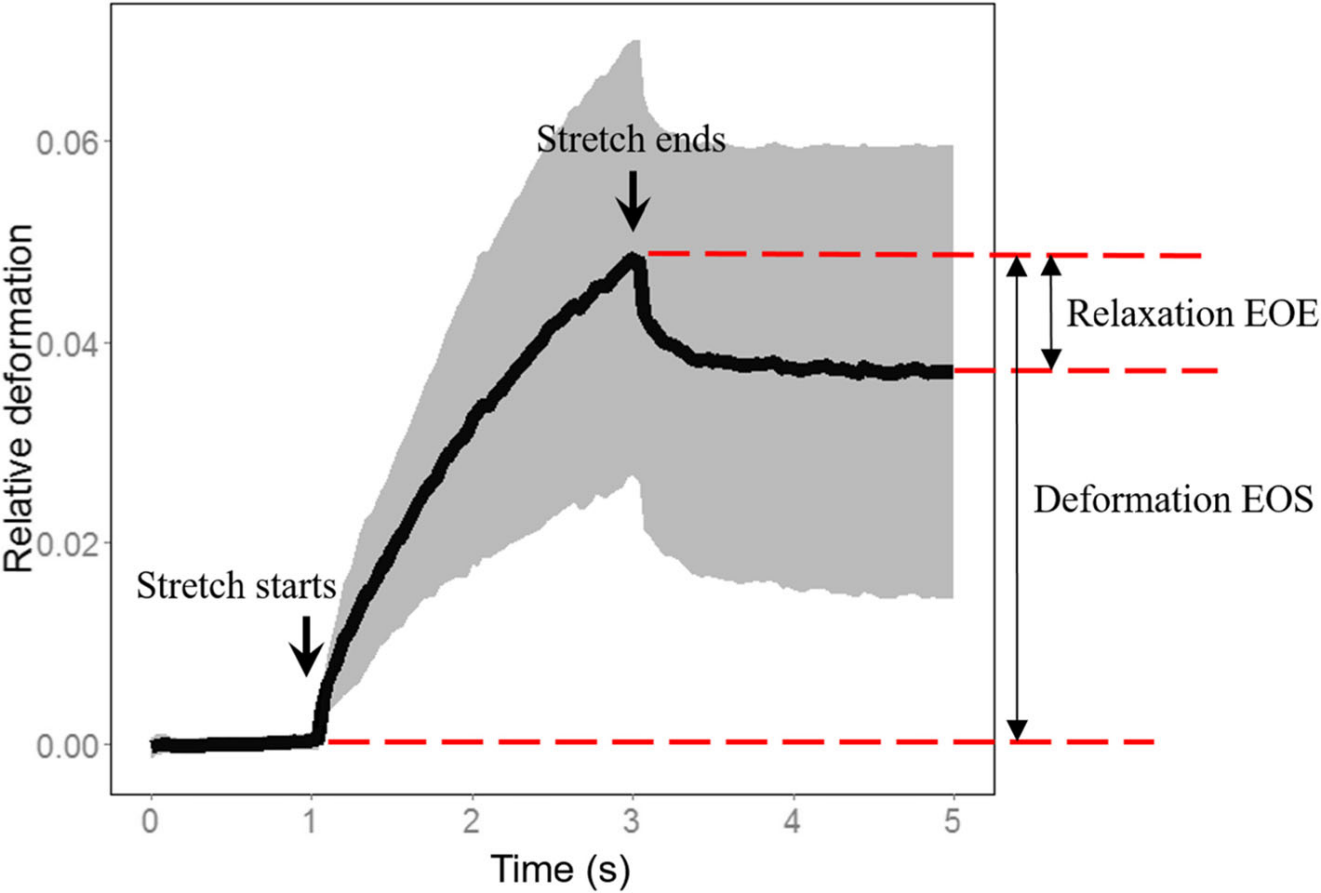
An illustration of the whole deformability data set of the optical stretcher. The two arrows show the starting and ending time point of stretch respectively. The thick black line shows average deformation of the length of long axis of cell over 130 cells. The gray area captures one standard deviation above and below the average. The two mechanical properties used in this paper (relaxation EOE and Deformation EOS) are illustrated. For both measurements, the deformation at the beginning of the experiment is subtracted. Hence the values of EOS are mostly positive and values of EOE mostly negative

We used a Microfluidic Optical Cell Stretcher to mechanically stretch individual cells from our breast cancer panel of cell lines and measure their stiffness (Kiessling et al. 2013; Lincoln et al. 2007a). Cells in suspension are not stimulated by their environment, and thus their cortical tension represents the cells’ mechanical “ground state”. Suspended single cells were trapped for 1 s and subsequently stretched for 2 s and then relaxed in trapping condition for another 2 s (Fig. 1). Images of cells were taken at the rate of 30 frames per second, and the length of the long axis was measured in each frame for each individual cell. In this paper, we use only two mechanical features calculated from these measurements: 1. Relative long axis deformation at the end of stretch (Deformation EOS), and 2. Relative long axis deformation after 2 s of relaxation (Relaxation EOE) (Fig. 1). The value of EOS is inversely proportional to the Young’s modulus, where higher EOS value indicates lower Young’s modulus (easier to stretch). On the other hand, EOE is a measure of the ability of a cell to restore its shape, where higher absolute value of EOE suggests greater ability to restore the original cell shape. The end-of-experiment deformation (EOE) can also be interpreted as the degree of cell plasticity of the cell under a given applied load or strain. This plasticity is in principle a coarse-grained property which contains contributions of the actin, microtubule and intermediate filament network (Kubitschke et al. 2017). Since both EOE and EOS are linear measurements, a two-fold change in the observed deformation corresponds to a two-fold change in elastic modulus.

In prior work where the mechanical measurements were parameterized by over 50 metrics, we identified deformation and relaxation as important independent determinants of cell mechanics (Kiessling et al. 2013). Together, these two features give a good estimation of the elastic property of a single cell.

Using this technique, we measured cells from our breast cancer cell panel used to study the EMT. MCF-10A is a non-tumorigenic breast epithelial cell line which is used as a control cell line. MDA-MB-231 and MDA-MB-436 are both triple negative breast cancer cell lines that are epithelial in nature. Both have metastatic potential, with MDA-MB-231 considered more aggressive than MDA-MB-436 (Bianchini et al. 2016).

### Two subgroups observed in MDA-MB-231 cells

**Fig. 2:**
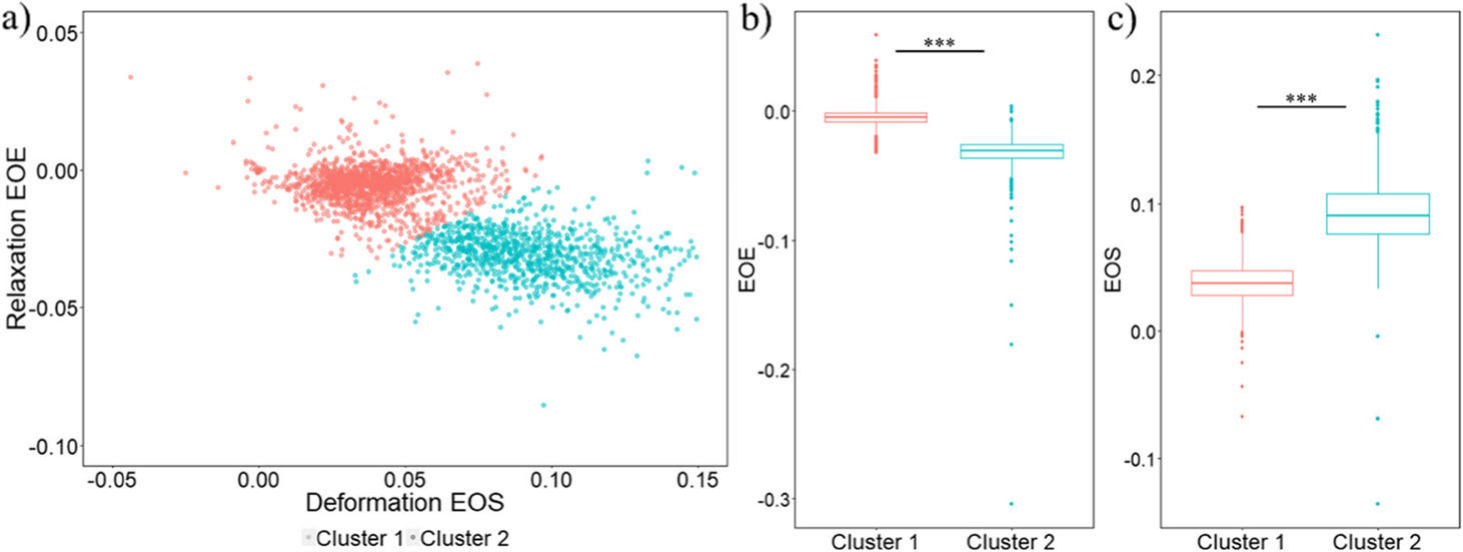
Two clusters of MDA-MB-231 cells are observed. **a** Scatterplot of Relaxation EOE vs Deformation EOS for MDA-MB-231 cells. The two subgroups are identified with the k-means clustering algorithm and labeled by different colors (red: cluster 1, blue: cluster 2). Negative EOS values in the plot can have two causes: first, strongly rotating cells that influence the shape detection; second, active contractions under force activation (data not shown). Similarly, a positive EOE values can indicate a strongly rotating cells or continued deformation during the relaxation phase. **b** Boxplot comparing relaxation at the end of experiment between cluster 1 and cluster 2 of MDA-MB-231 cells (*p* value < 0.001). **c** Boxplot comparing deformation at the end of stretch between the two subgroups of MDA-MB-231 cells (*p* value < 0.001)

**Fig. 3:**
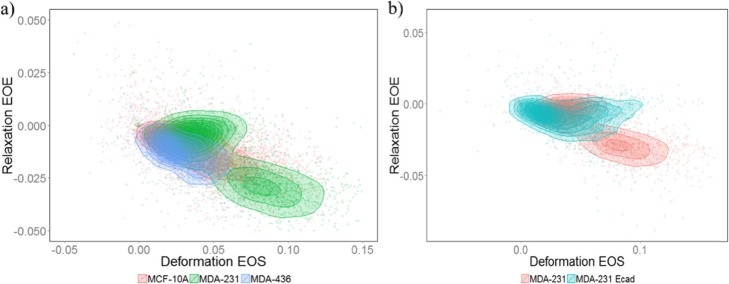
MCF-10A, MDA-MB-436 and E-cadherin labeled MDA-MB-231 cells all overlap with cluster 1 (the less elastic group) in unlabeled MDA-MB-231 cells. **a** Scatterplot of Relaxation EOE vs Deformation EOE for MCF-10A (red), MDA-MB-231 (green) and MDA-MB-436 (blue) cells. **b** Scatterplot of Relaxation EOE vs Deformation EOE for E-cadherin labeled (blue) and unlabeled (red) MDA-MB-231 cells

We first identified two subgroups within MDA-MB-231 cells. One subgroup (cluster 2, Fig. 2) exhibited higher deformations at the end of stretch (EOS) and higher absolute values of relaxation at the end of experiment (EOE) than the other subgroup (cluster 1) (Fig. 2). Higher absolute values of both EOS and EOE indicate that cells in cluster 2 are softer and more elastic (easier to stretch and easier to restore original shape) compared to cluster 1, which overlaps with MDA-MB-436 and MCF-10A cells (Fig. 3a).

### The more elastic group does not exist in MDA-MB-231 cells labeled for E-cadherin

Cadherins are responsible for cell-cell binding. E-cadherins are expressed in normal epithelial cells, while in mesenchymal carcinoma cells it is mainly N-cadherins. In our experiments, we also measured mechanical properties of MDA-MB-231 cells that were labeled with E-cadherin antibodies in order to activate extracellular binding sites. Since this is a mesenchymal-like cell line we found a low level of E-cadherin expression, as has also been quantified elsewhere (Pawlizak et al. 2015). In spite of the low expression levels, we observed a different stretching and relaxation behavior in the E-cadherin labeled and non-labeled MDA-MB-231 cells. E-cadherin labeled MDA-MB-231 cells only formed one cluster instead of the two clusters observed in unlabeled MDA-MB-231 cells. The labeled 231 cells overlap with the less elastic and less relaxing subgroup of MDA-MB-231 cells (cluster 1, Fig. 3b). Activation of the E-cadherin receptor by binding of the antibody leads to cadherin clustering and E-cadherin binding to the actin cortex, which upregulates the actin polymerization and cross-linking of the cytoskeleton (Perez-Moreno and Fuchs 2006). The decrease in deformation found in cluster 1 cells compared to cluster 2 cells is consistent with this change in mechanics due to E-cadherin activation since the elastic storage modulus strongly depends on crosslinking density and dynamics (Gardel et al. 2004; Lieleg et al. 2010; Strehle et al. 2011; Schnauß et al. 2016). In addition, the decreased cell relaxation of the cluster 1 subpopulation could also be explained with upregulated actin nucleation and aggregation while a destabilization of the microtubular cytoskeletal backbone may further result in a lack of relaxation and increased plasticity (Kubitschke et al. 2017).

### MDA-MB-231 and MDA-MB-436 cells are more different from each other than from MCF-10A cells

**Fig. 4:**
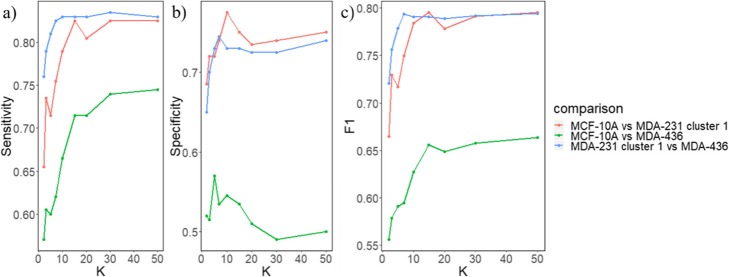
Pairwise k-NN classification results show that MDA-MB-231 and MDA-MB-436 cells are more different from each other than from MCF-10A cells. **a** Sensitivity (true positive rate) for the three comparisons versus different values of k. **b** Specificity (true negative rate) for the three comparisons versus different values of k. **c** F1 score for the three comparisons versus different values of k

**Fig. 5:**
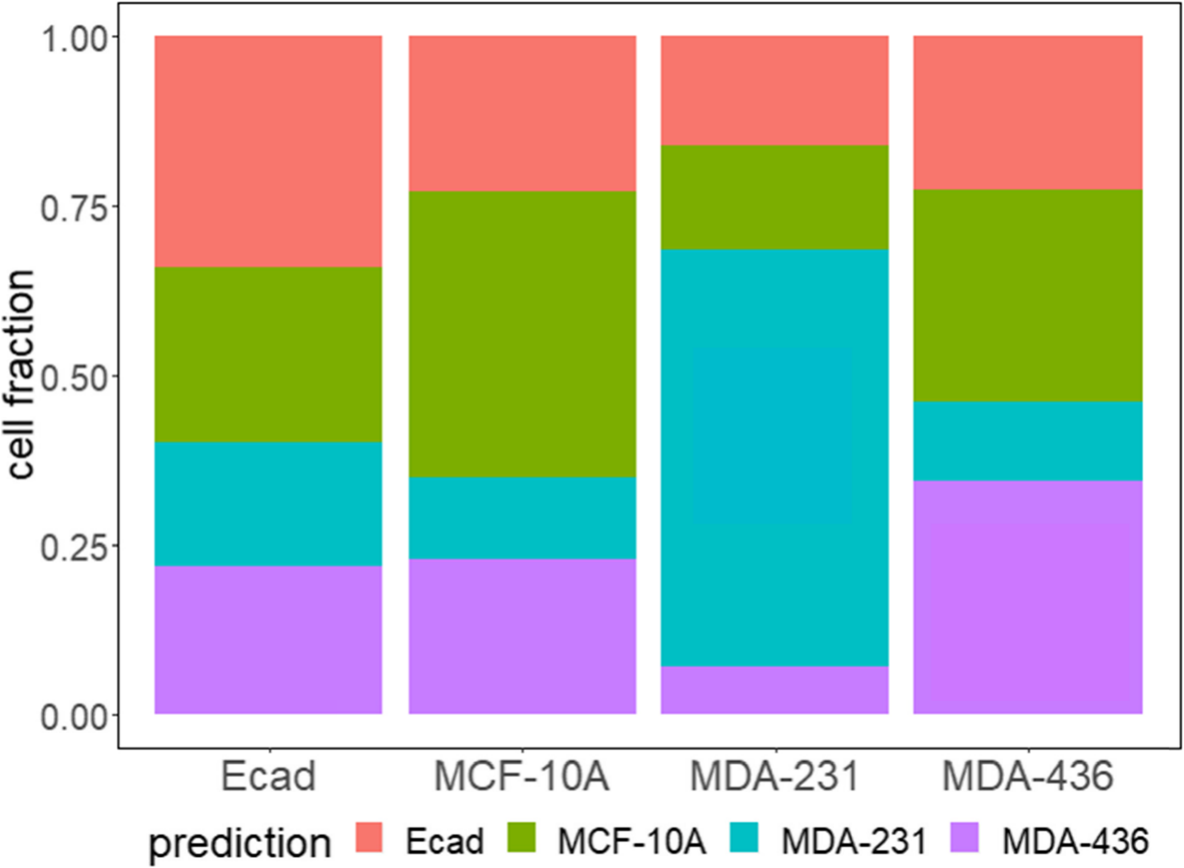
k-NN classification results of E-cadherin labeled MDA-MB-231 cells (Ecad), MCF-10A cells, cluster 1 in MDA-MB-231 cells and MDA-MB-436 cells, with k = 10

While we showed above that cluster 1 of MDA-MB-231 cells greatly overlaps with MCF-10A and MDA-MB-436 cells, these three cell lines may still be separable at the single cell level. Since both MDA-MB-436 and MDA-MB-231 cell lines have a malignant mesenchymal-like phenotype, it is reasonable to expect they would be more similar to each other comparing to the epithelial-like MCF-10A cell line. To separate the cell phenotypes, we applied a k nearest neighbors (k-NN) algorithm for a pairwise classification of the three phenotypes. We first divided the cells into two groups: train and test. Phenotype labels were provided for cells in the training group but not for the test group. Then, given the position of a single cell in the test group, k-NN identifies its nearest k neighbors within the training group. The k neighbors then take a “vote” with their phenotype, and the cell from test group is assigned to the phenotype that has the highest number of votes. After classification, we calculate the sensitivity (true positive rate), specificity (true negative rate), and F1 score (a measure of classification result, the higher the score the better the classification; the maximum F1 score is 1) for each pair of classification. We found that classification between MCF-10A and MDA-MB-436 cells has the lowest sensitivity, specificity and F1 score regardless of the value of k (green line in Fig. 4). On the other hand, classification between cluster 1 of MDA-MB-231 and MDA-MB-436 cells had the highest level of F1 scores (blue line in Fig. 4c) – which was even higher than the classification between MCF-10A and MDA-MB-231 cells (red line in Fig. 4c) for most values of k. Similar results were obtained with a different classification algorithm (SVM), where the classification between MDA-MB-231 and MDA-MB-436 cells also had the highest F1 value (Table 1). SVM takes a different approach in classification and aims to find the linear plane that best separates two groups to classify. In addition, when all four phenotypes were classified simultaneously, MDA-MB-436 cells were less likely to be miss-classified as MDA-MB-231 cells than as MCF-10A cells and vice versa (Fig. 5). Together, these results suggest that cells in cluster 1 of MDA-MB-231 are more different from MDA-MB-436 cells than from MCF-10A cells despite that MDA cells are phenotypically considered to be mesenchymal-like and MCF cells to be epithelial.

**Table 1:** Pairwise classification results by support vector machine (SVM)

	Sensitivity	Specificity	F1
MCF-10A (positive) vs MDA-MB-231 cluster 1 (negative)	0.71	0.72	0.71
MCF-10A (positive) vs MDA-MB-436 (negative)	0.58	0.66	0.60
MDA-MB-231 cluster 1 (positive) vs MDA-MB-436 (negative)	0.74	0.79	0.76

## Discussion

Mechanical properties of tumors cells may be important markers for the metastatic potential of tumors. Studies have shown that metastatic tumor cells are on average softer than non-metastatic ones (Xu et al. 2012; Guck et al. 2005; Fritsch et al. 2010; Alibert et al. 2017; Mierke 2015). In this paper, we illustrate the heterogeneity of tumor cell stiffness both within and between cell lines. Based only on mechanical properties, we show that there are two distinct clusters within MDA-MB-231 cells. Cluster 1 greatly overlaps with MCF-10A and MDA-MB-436 cells, while cells in cluster 2 are softer and more elastic (easier to deform and restore to original shape). In addition, we found that the two malignant epithelial cell lines, MDA-231 and MDA-436, are more distinct from each other in their mechanical phenotype than from the non-tumorigenic MCF-10A cell line.

Our findings of mechanical heterogeneity within the MDA-MB-231 cell line complement prior findings indicating that the molecular single cell characteristics of MDA-MB-231 cells are also heterogeneous. For example, it has been shown that there are two distinct subgroups of MDA-MB-231 cells which differ significantly in the cell surface density of various cytokine receptors (CCR5, CXCR3, CXCR1) (Norton et al. 2015). In particular, CXCR3 was found to be overexpressed in metastatic tumor cells, and drugs targeting CXCR3 decreased tumor cell migration (Zhu et al. 2015). To link our observations of mechanical heterogeneity with molecular heterogeneity directly, future studies can combine the optical stretching with fluorescence imaging.

We also identified heterogeneity among different triple negative breast cancer (TNBC) cell lines, i.e. we found that MDA-MB-231 and MDA-MB-436 cells are quite distinct from each other, even more so than from the non-tumorigenic MCF-10A cell line. This finding is consistent with the perspective of the classical clonal evolution model, assuming the epigenetic and (more importantly) the phenotypic characteristics of normal breast tissue are similar among all women. Thus, both patients from which the MDA-MB-231 and MDA-MB-436 cell lines are derived, had initially breast tissue which is very similar to the MCF-10A tissue. From this healthy starting population of cells, different paths can be taken to reach a metastatic phenotype. In fact, an extensive gene similarity analysis based on The Cancer Genome Atlas (TCGN) revealed that on average approx. 40% of tumors of a given site, e.g. breast cancers, are likely genetically closer to tumors from other sites than to tumors of the same origin (Heim et al. 2014; Andor et al. 2016). It seems actually unlikely that two completely different patients accumulate the exact same cancer cell phenotype with the same optical stretching characteristics.

In addition, our findings may have important clinical implications. Patients with triple negative breast cancer are currently considered to have a very poor prognosis (Bianchini et al. 2016; Lehmann and Pietenpol 2014; Denkert et al. 2017). However, there has been an emerging trend to regard TNBC as a heterogeneous group of patients with varying prognosis (Bianchini et al. 2016; Lehmann and Pietenpol 2014; Denkert et al. 2017). Furthermore, TNBCs can have very different molecular characteristics, potentially rendering some tumors more suitable to targeted therapies (Bianchini et al. 2016; Lehmann and Pietenpol 2014; Denkert et al. 2017). It is of paramount clinical importance to identify those patients. The present data is exciting in that it shows that two TNBC cell lines (which would be put into one prognostic basket clinically) are indeed very different. It is intriguing to speculate whether optical stretching analysis could be used to differentiate those TNBC cases with a better prognosis (i.e. a lower rate of relapse and distant metastasis) from those with a worse prognosis.

Moreover, our findings on the inter-cell-line heterogeneity is an indication that average based analysis methods could oversimplify tumor cell data. For example, MCF-10A, MDA-MB-436 and cluster 1 of MDA-MB-231 cells are mechanically similar to each other with probably minor difference in the average values (Fig. 3a). However, when classified with a more sophisticated algorithm like k-NN, reasonably good classification accuracy can be achieved. That is to say, even though cells from the three cell lines overlap on average, locally cells from a certain cell line are closer to cells from the same cell line than from other cell lines.

Lastly, our studies of E-cadherin labeled MDA-MB-231 cells reveal that antibody labeling can alter the mechanical phenotype significantly. We reason that this is because binding of the antibody to the E-cadherin receptor simulates cell-cell binding, which causes cadherin clustering and stimulates the actin cortex bound to cadherin. This is a good example of how antibody labeling may change the properties of cells, and how antibodies could provide insights into the changes in cancer cell behavior in response to their tumor microenvironment. Further experiments are needed to validate and provide molecular evidence for the role of E-cadherin antibody treatment in altering the mechanical phenotype of MDA-MB-231 cells.

## Conclusion

In conclusion, we illustrated heterogeneity in cellular mechanical properties within and between cell lines. Future studies should examine how changes in chemokine receptor expression correlate with tumor cell stiffness. Additional investigations are needed to determine how mechanical properties of cancer cells could help identify distinct prognostic subgroups of triple negative breast cancer patients.

## Methods and materials

### Experimental procedures

The general setup of the optical stretcher (OS) is described in (Lincoln et al. 2007b) with additional improvements to the microfluidics, the computer̵-controlled stretching processes, and the thermally controlled stage described in detail in (Lincoln et al. 2007b; Guck et al. 2001; Schmidt et al. 2015). The mechanical properties of cells were determined by guiding the cell suspension into the automated microfluidic OS where single cells are consecutively trapped and stretched. The cells are trapped at 100 mW for 1 s and the cell radius along the laser axis is determined. The cell is afterwards stretched at 875 mW for 2 s. The cells are allowed to relax for 2 s after stress cessation. A microscope-mounted camera takes images at 30 frames per second during the whole stretching process. Afterwards, an edge detection algorithm is used to extract cell shape and cell parameters and to sort out pathological cell (e.g. dead cells).

### Cell culture and medium

MCF-10A cells (Cat.No. CRL-10317, ATCC) were cultured in DMEM/Ham’s F12 medium containing l-glutamine (Cat.No. FG 4815, Biochrom) supplemented with 5% horse serum (Cat.No. 12449C, SAFC), 20 ng/ml human epidermal growth factor (Cat.No. E9644, Sigma-Aldrich), 10 μg/ml insulin (Cat. No.I9278, Sigma-Aldrich), 100 ng/ml cholera toxin (Cat.No. C8052, Sigma-Aldrich), 500 ng/ml hydrocortisone (Cat.No. H0888, Sigma-Aldrich) and 100 U/ml penicillin/streptomycin (Cat.No. A 2213, Biochrom).

MDA-MB-231 and MDA-MB-436 cells were cultured in DMEM containing 4.5 g/l glucose, l-glutamine (Cat.No. FG 0435, Biochrom) supplemented with 10% fetal bovine serum (Cat.No. S 0615, Biochrom) and 100 U/ml penicillin/streptomycin.

All cell lines were incubated at 37 °C in a 95% air and 5% CO2 atmosphere. The culture medium was changed every 2 to 3 days and cells were passaged every 4 to 5 days. To detach the cells, a PBS solution containing 0.025%(w/v) trypsin and 0.011%(w/v) EDTA (Cat.No. L 2113, Biochrom) was applied for several minutes.

### Data analysis

The two clusters of MDA-MB-231 cells were identified using the *kmeans*() function in *R* (version 3.0.3) with 2 centers, 1000 iterations and 50 random initial conditions. For kNN classification, 1200 cells were first randomly selected from each cell line. From the 1200 cells, 200 were randomly selected as testing set and the remaining 1000 were used as training set for each cell line. The classification was done separately for each pair of cell line using the *knn*() function in R with 8 different values of *k* (2, 3, 5, 7, 10, 15, 20, 50). Similarly, simultaneous classification of the three cell lines were done. After classification, a false positive rate was calculated as FPR = (false positives) / (false positives + true positives), and a false negative rate was calculated as (FNR) = (false negatives) / (false negatives + true negatives). Finally, pairwise support vector machine (SVM) classifications were done based on all 1200 randomly selected cells using the *ksvm*() function with linear kernel and *C* = 10 in the *R* package *kernlab*. All plots were made with the ggplot2 package in *R*. The dataset is normalized to zero mean and unit variance before the aforementioned analysis.

## Data Availability

Data is available upon request.

## Abbreviations

EOE: End of experiment deformation
EOS: End of stretch deformation
FNR: False negative rate
FPR: False positive rate
kNN: k nearest neighbors
OS: Optical stretcher
SVM: Support vector machine
TCGN: The Cancer Genome Atlas
TNBC: Triple negative breast cancer
